# The microbiota of pregnant women with SARS-CoV-2 and their infants

**DOI:** 10.1186/s40168-023-01577-z

**Published:** 2023-06-26

**Authors:** Heidi K. Leftwich, Daniela Vargas-Robles, Mayra Rojas-Correa, Yan Rou Yap, Shakti Bhattarai, Doyle V. Ward, Gavin Fujimori, Catherine S. Forconi, Tracy Yeboah, Acara Carter, Alyssa Kastrinakis, Alison M. Asirwatham, Vanni Bucci, Ann M. Moormann, Ana Maldonado-Contreras

**Affiliations:** 1 Department of Obstetrics and Gynecology, Division of Maternal-Fetal Medicine, University of Massachusetts Memorial Health, University of Massachusetts Chan Medical School, Worcester, MA USA; 2 Department of Microbiology and Physiological Systems, Program of Microbiome Dynamics, University of Massachusetts Chan Medical School, Worcester, MA USA; 3 Department of Medicine. Division of Infectious Diseases and Immunology, University of Massachusetts Chan Medical School, Worcester, MA USA

## Abstract

**Background:**

Infants receive their first bacteria from their birthing parent. This newly acquired microbiome plays a pivotal role in developing a robust immune system, the cornerstone of long-term health.

**Results:**

We demonstrated that the gut, vaginal, and oral microbial diversity of pregnant women with SARS-CoV-2 infection is reduced, and women with early infections exhibit a different vaginal microbiota composition at the time of delivery compared to their healthy control counterparts. Accordingly, a low relative abundance of two *Streptococcus* sequence variants (SV) was predictive of infants born to pregnant women with SARS-CoV-2 infection.

**Conclusions:**

Our data suggest that SARS-CoV-2 infections during pregnancy, particularly early infections, are associated with lasting changes in the microbiome of pregnant women, compromising the initial microbial seed of their infant. Our results highlight the importance of further exploring the impact of SARS-CoV-2 on the infant’s microbiome-dependent immune programming.

Video Abstract

**Supplementary Information:**

The online version contains supplementary material available at 10.1186/s40168-023-01577-z.

## Background

Large fractions of microbes that newly colonize a newborn come from their mother [1–7]. This newly acquired microbiome exerts marked effects on the immune programming of infants with long-term health consequences, including susceptibility to infections or chronic inflammatory diseases and reduced vaccine efficacy [8–15]. Particularly, epidemiological and mechanistic studies in animal models have established that microbial dysbiosis in early life influences disease pathogenesis via changes in immune system maturation [12–14]. While the microbiome and the immune system dynamics can converge later in human life [16], many acute and chronic diseases have now been associated with changes in the oral and gut microbiomes, and there is evidence of the complex interplay between the immune system, systemic physiology, and the microbiome in various health conditions. Therefore, this window of opportunity for microbiome inoculation, either renders infants with a healthy immune system or alternatively establishes a divergent path leading to severe immune-mediated disease susceptibility [17–25]. The impact of SARS-CoV-2 infections on pregnant women and their offspring microbiotas has still been explored.

Previous studies have found that SARS-CoV-2 patients with severe symptoms display a dysbiotic microbiome (reviewed in [26]. Pregnant women with SARS-CoV-2 infection are at a higher risk of experiencing severe symptoms, which can result in increased risk of maternal death (odds ratio 6.09), mechanical ventilation (odds ratio 2.61), admission to intensive care units (odds ratio 5.41), preterm birth (odds ratio 1.57), cesarean delivery (odds ratio 1.17), pneumonia (risk rate 23.5), and thromboembolic disease (risk rate 5.5) [27, 28].

To our knowledge, only two studies have characterized the microbiotas of pregnant women with SARS-CoV-2 infection. The first study, conducted in Spain, reports an increased abundance of *Bacteroidales* in the nasopharyngeal swabs of pregnant women with active SARS-CoV-2 infection compared to healthy controls [29]. In the second study, conducted in Mexico, authors report differences in the gut microbiota of SARS-CoV-2-positive pregnant women and their infants compared to the pregnant women with no evidence of SARS-CoV-2 viral particles in stool [30]. Most of the pregnant women included in the latter study were negative for SARS-CoV-2 on nasopharyngeal swabs and asymptomatic at the time of sample collection, suggesting past SARS-CoV-2 infection.

Besides gut microbiota, the vaginal and oral microbiotas of pregnant women represent the initial seed of the infants’ gut microbiota if born vaginally [1, 3]. There is a paucity of research in understanding the impact of SARS-CoV-2 infection at different stages of pregnancy on the initial seed of the infant’s microbiota (i.e., gut, vaginal, and oral microbiotas of pregnant women). Hence, the objective of this study is to determine whether infection by SARS-CoV-2 during pregnancy, either at early or late stages of pregnancy or an active infection at delivery results in gut, vaginal, and oral microbiota changes that are passed onto the offspring.

## Methods

### Patient recruitment

We enrolled pregnant women and their newborns delivered at the University of Massachusetts Memorial Hospital between April 2020 and August 2021. A total of 88 pregnant women, 62 with positive SARS-CoV-2 diagnosis, 26 with a negative SARS-CoV-2 diagnosis, and 68 newborns (2 sets of twins) were recruited. Participants were classified as having had SARS-CoV-2 infection by clinical PCR-positive viral DNA diagnostic test at any time during pregnancy or as healthy controls if no positive diagnostic test was either listed in their medical record or reported by the patients, and they tested negative upon admission to labor and delivery, as per hospital protocol. We also, defined negatives to SARS-CoV-2 by ELISA, as previously defined by us [31]. Briefly, a maximum specificity threshold was established based on a cutoff at 100% specificity for the NP IgG assessed on pre-pandemic samples collected from 96 adult healthy individuals. The cutoff is an OD value of 0.57 and represents the highest OD value for NP IgG in those pre-pandemic adult healthy individuals [31].

SARS-CoV-2-positive participants were further sub-classified by the time of SARS-CoV-2 diagnosis in “early” (1st–2nd trimester), “late” (3rd trimester), or “active” (SARS-CoV-2 positive at delivery) group. Mother–infant dyads were incomplete when parents only consented to the collection of either mother or infant samples. This cohort was recruited under the COVID-19 Analysis on Perinatal Specimens Related to Exposure (CARES) protocol (docket # H00020145), with 10 healthy pregnant women and their newborns recruited as part of our ongoing MELODY trial (docket # H00016462) [32]. The Institutional Review Board at the University of Massachusetts Chan Medical School approved both studies. Informed consent was obtained from all study participants or their health care proxy using REDCap digital signatures to reduce the potential for patient–staff transmission of SARS-CoV-2 or based on the remote data collection study design for the MELODY project. Participants were asked to consent separately to each of these sample collections, and therefore, not all participants had samples in each cohort.

### Sample collection

After obtaining consent, all samples were collected by the attending or resident physician or nurse caring for the patient at delivery time. From pregnant women, we obtained anal, oral, and vaginal swabs before delivery. For anal samples, a sterile swab (Sterile Flocked Swab. Puritan Medical Products Company LLC, ME, USA) was inserted 1 to 2 in into the anus to obtain gut material from pregnant women prior to delivery. Oral swabs were obtained using the OMNIgene•ORAL (DNAGenotek™, Canada) following the manufacturer instructions. Briefly, the oral mucosa was sampled from the tongue for 30 s. We used the OMNIgene•VAGINAL (DNAGenotek™, Canada) to obtain vaginal samples prior to delivery. Specifically, a sterile swab was inserted 1 to 2 in into the vagina and rotated in circles along the vaginal walls for 20 s. After swabbing, both oral and vaginal swabs were inserted into their respective tubes containing a DNA/RNA stabilizer buffer. For antibody assays, we collected cord blood after delivery, namely, 5 cc of blood were withdrawn or drained into an EDTA tube. Plasma was separated from peripheral blood cell pellet by centrifugation (10 min at room temperature), and aliquots were stored at − 20 °C until thawed for antibody testing. For the newborns, we collected samples 1–2 days after delivery, namely, a diaper with the meconium as previously described and an oral swab as described above.

### Clinical data

All clinical data were obtained retrospectively by reviewing the electronic medical records of each participant following delivery.

### Nucleic acid isolation

To minimize the risk of SARS-CoV-2 infection, oral samples were inactivated at 65–70 °C for 1 h, as demonstrated elsewhere [33]. Then, samples were pre-treated with Proteinase K (Cat # P8107S, New England Biolabs, MA, USA) and incubated for 2 h at 50 °C and subsequently used for nucleic acid isolation. Nucleic acid isolation of oral samples and mother’s anal swabs were performed using the ZymoBIOMICS DNA/RNA Miniprep Kit (Cat # D7003/D7003T, Zymo Research, CA, USA) following the manufacturer recommendations for parallel isolation of DNA and RNA. Nucleic acid from the meconium of the newborns was isolated using DNeasy Power Soil Pro kit (Cat # 47,016, Qiagen, CA). Due to the tar-like consistency of the meconium, a combination of bead-beating, 30 min of heating at 80 °C, and only 90–100 mg of sample was used for the initial lysis step.

### Microbiome profiling

The *16S rRNA* gene was sequenced following methods previously described [33] using the 341F and 806R universal primers to amplify the V3**–**V4 region. The 300 nt paired-end sequences were generated on the Illumina MiSeq platform. Replicate reactions were performed for each sample and the read data were merged for analysis. Only, forward *16S rRNA* gene MiSeq-generated amplicon sequencing reads were dereplicated, and sequence variants (SV), also known as amplicon sequence variants (ASV), were inferred using DADA2 [33]. We obtained on average 57,428 (± 43,107) sequences per sample. Generated sequences were deposited in the NCBI database, BioProject ID: PRJNA871082. Potentially chimeric sequences were removed using consensus-based methods. Taxonomic assignments were made using BLASTN against the NCBI refseq RNA database combined with GreenGenes, the Human Oral Microbiome Database, and previously used cervicovaginal microbiome *16S rRNA* reference sequences from NCBI [34]. These files were imported into R and merged with a metadata file into a single Phyloseq object. Samples were rarefied at 4085 sequences per sample. The rarefaction threshold was selected arbitrarily to include a greater number of samples with an adequate number of sequences for analysis while also minimizing the loss of samples with lower sequence counts. Feature table at the SV level was used for alpha and beta diversity analyses and random forest classifications.

### Community state types (CSTs)

Each woman’s sample was classified into CST following the protocol of the VALENCIA program [35] with Python 3 (https://github.com/ravel-lab/VALENCIA). Input data was formatted using local scripts.

### Random forest classification (RFC)

RFC was used to find microbiome and clinical variables that could predict SARS-CoV-2 infection. First, the feature selection was run, in which the wrapper Boruta [36] is used to identify a subset of covariates that is predictive of the outcome, followed by RFC utilizing only the Boruta-selected subset. For the RFC interpretation, the models were entered into the Local Interpretable Model-agnostic Explanation (LIME) toolbox [37]. LIME allows identified human-interpretable logical rules on the microbiome to distinguish between patients with different outcomes. We performed RFC using microbial features at the SV taxonomic level. To reduce zero inflation, we only include bacteria that have an average abundance above 1E − 4 across all samples for RFC.

### Antibody ELISA

Antibodies against the receptor-binding domain (RBD) of the SARS-CoV-2 spike protein were measured by ELISA following published methods [38]. In brief, an IgG antibody against the nucleocapsid protein (NP, gift from Lisa Cavacini, UMass-Biologics) was used at 0.5 μg/mL and incubated with plasma at a 1:100 dilution. Optical density (OD) was measured at 450 and 570 nm on the SpectraMax iD5 ELISA plate reader (Molecular Devices) using SoftMax Pro software (version 7.1, Molecular Devices). For the positive antibody control, monoclonal therapeutic CR3022 IgG antibody (gift from Lisa Cavacini, UMass-Biologics) was diluted from a concentration of 2.5 μg/ml in dilution buffer to 12 two-fold serial dilutions to generate the standard control curve [39]. The 570 nm OD was subtracted from the 450 nm OD for the final OD value. Antibody levels were used as a continuous variable in the analysis.

### Statistical analysis

Fisher’s exact test and Kruskal–Wallis or Wilcoxon–Mann–Whitney test were used to evaluate differences in demographics among pregnant women with different SARS-CoV-2 statuses by pregnancy stage (SARS-CoV-2 positive/HC and early/late/active SARS-CoV-2 infection/HC). For the CST analysis, we performed Fisher’s exact test, as described above and also stratified by each CSTs separately. For the microbiota diversity analyses, comparisons were performed between groups at SV level using multiple functions from Phyloseq v1.19.1 package [40] in R [41]. Microbial alpha diversities were measured using Shannon index or Chao1 estimator. Linear regression models (LM) were run with “lm” base R function. Alpha diversity metrics were set as the dependent variables, while SARS-CoV-2 infection or time of SARS-CoV-2 infection, race, antibiotic use, mother’s age, pre-pregnancy body mass index (BMI), gestational diabetes, and delivery mode (the last one only for infant samples) as the independent variables. Best-fitted models were chosen using the “step” R function. The “emmans” function [42] was used as a pairwise analysis since it computes contrasts, trends, and comparisons of slopes among groups. Beta diversity was evaluated using Bray–Curtis dissimilarity and Sørensen index; the former considers the SV abundance while the latter only the incidences. The same independent variables as for alpha diversity analysis were included in the beta diversity analyses using the non-parametric permutational multivariate analysis of variance (PERMANOVA) [43] with “adonis2” from vegan package [44], and pairwise analyses were performed with “pairwiseAdonis” [45]. PERMANOVA allows comparing variance between groups to the variance within groups (spatial location differences). Sample dispersion was also evaluated using PERMDISP2 procedure [46] with “betadisper” function, which executed the analysis of multivariate homogeneity of group dispersions (variances), adjusting for the different sample sizes to avoid bias. Association between each microbial relative abundance (SV taxa collapsed at the genus level, as previously performed [47]) and fecal calprotectin levels was assessed by the spearman correlation test with “cor.test” function on base R. The correlation analysis excluded genera with a mean abundance of < 0.1% or more than 80% zero values, as previously performed [47]. *P* values were all adjusted with the false discovery rate method using the “p.adjust” function from R base. Plots were generated using the "ggplot2" [48] package and base R functions and edited in Adobe Illustrator [49].

## Results

### Participant description

A total of 88 participants were included in the study. Sixty-two participants had a confirmed PCR-SARS-CoV-2 infection sometime during their pregnancy while 26 were healthy throughout pregnancy with no antibodies for SARS-CoV-2. Participants had an average age of 31.7 years, and a pre-pregnancy BMI of 30.7, consistent with a diagnosis of obesity. In fact, 41.0% of participants were obese by pre-pregnancy BMI, while 16.2% exhibited a normal BMI. Importantly, obesity did not significantly differ between infected and healthy controls (Table 1, *P* = 0.321).

**Table 1:** Demographic and clinical variables of the pregnant participants included in the study. SARS-CoV-2-positive participants and SARS-CoV-2-negative (healthy controls) were recruited at the University of Massachusetts Memorial Hospital from April 27 to June 10, 2020. Additionally, SARS-CoV-2 negative (healthy controls) were recruited nationwide prior to the COVID-19 pandemic

**Demographics and clinical variables**	**SARS-CoV-2-positive pregnant women (*N* = 62)**	**Healthy controls pregnant women (*N* = 26)**	**Total (*N* = 88)**	***P value***^a^
Age				0.032
Mean (SD)	31.0 (6.24)	33.4 (4.96)	31.7 (5.97)	
Body mass index				
Pre-pregnancy				0.473
Mean (SD)	31.2 (6.93)	29.5 (7.33)	30.7 (7.05)	
Pre-pregnancy category				0.321
Underweight	1 (1.6%)	1 (3.8%)	2 (2.3%)	
Normal	11 (17.7%)	4 (15.4%)	15 (17.0%)	
Overweight	20 (32.3%)	13 (50.0%)	33 (37.5%)	
Obese	30 (48.4%)	8 (30.8%)	38 (43.2%)	
Race				0.048
Non-Hispanic White	29 (46.8%)	18 (69.2%)	47 (53.4%)	
Hispanic or Latino	24 (38.7%)	2 (7.7%)	26 (29.5%)	
Non-Hispanic Black	7 (11.3%)	4 (15.4%)	11 (12.5%)	
Non-Hispanic Asian	2 (3.2%)	2 (7.7%)	4 (4.5%)	
SARS-CoV-2 comorbidities				
Type 2 diabetes	4 (6.5%)	2 (7.7%)	6 (6.8%)	0.889
Cardiovascular disease	12 (19.4%)	4 (15.4%)	16 (18.2%)	0.669
Pregnancy outcomes				
Vaginal delivery	43 (69.4%)	16 (61.5%)	59 (67.0%)	0.621
Preeclampsia	9 (14.5%)	2 (7.7%)	11 (12.5%)	0.549
Preterm (≤ 37 weeks)	14 (22.6%)	3 (11.5%)	17 (19.3%)	0.181
Gestational diabetes	13 (21.0%)	3 (11.5%)	16 (18.2%)	0.502
Antibiotic during delivery	24 (38.7%)	12 (46.2%)	36 (40.9%)	0.724
Antibiotic before delivery	5 (8.1%)	0 (0%)	5 (5.7%)	0.341
Vaccinated against SARS-CoV-2	1 (1.6%)	9 (34.6%)	10 (11.4%)	3.00E − 05

Wilcoxon–Mann–Whitney test for continuous variables

^a^Fisher's exact test for categorical variables

In this cohort, age and race were significantly different between healthy and SARS-CoV-2-positive pregnant individuals. Particularly, healthy pregnant individuals were on average older and mostly non-Hispanic White (Table 1, *P* < 0.048). As most of our recruitment occurred before vaccines against SARS-CoV-2 became available, only a fraction of the participants was vaccinated at the time of sample collection. The COVID-19 vaccination rate was higher in healthy participants than SARS-CoV-2-positive participants (Table 1, *P* = 3.00E − 5). There were no statistical differences in any of the demographics or clinical variables accounting by the time of SARS-CoV-2 infection (Table S2, *P* = 0.361).

The infants recruited for the study were 57.4% female, with a mean of 3270 g of body weight, and 67.6% were delivered vaginally. A total of 14.7% of infants were admitted to the newborn intensive care unit mostly due to pre-term birth (*P* < 0.0001) and not associated with SARS-CoV-2 infection during pregnancy (*P* = 0.278) or with active infections at delivery (*P* = 0.436). There were no differences in demographic or anthropometrics measurements between infants born to SARS-CoV-2-positive or healthy women or between infants grouped by time of infection (Table 2, Table S2, *P* > 0.050).

**Table 2:** Demographic and clinical descriptions of infants included in the study. Infants born to SARS-CoV-2-infected pregnant participants and healthy controls were recruited at the University of Massachusetts Memorial Health from April 27 to June 10, 2020. Additional infants born to healthy controls were recruited nationwide prior to the COVID-19 pandemic

**Demographics and clinical variables**	**Infants born to SARS-CoV-2-positive pregnant women (*N* = 47)**	**Infants born to healthy controls pregnant women (*N* = 21)**	**Total (*N* = 68)**	***P value***^a^
Gender				0.282
Female, *N* (%)	29 (61.7%)	10 (47.6%)	39 (57.4%)	
Infant weight (g)				0.518
Mean (SD)	3200 (638)	3420 (444)	3270 (590)	
Delivery mode				0.909
Vaginal, *N* (%)	32 (68.1%)	14 (66.7%)	46 (67.6%)	
NICU admission,* N (%)*	8 (17.0%)	2 (9.5%)	10 (14.7%)	0.423

^a^Fisher’s exact test

### SARS-CoV-2 infection is associated with changes in the microbiota composition of pregnant women

We evaluated the microbial diversity of pregnant women with SARS-CoV-2 infection during pregnancy and compared it to healthy controls; we also compared microbial diversity by the time of SARS-CoV-2-positive diagnosis. Regardless of the timing of the SARS-CoV-2-positive diagnosis, all the samples were collected from pregnant women at delivery admission, prior to delivery. Alpha and beta diversity assessments and random forest classification (RFC) were performed, including potential cofounder variables such as race, antibiotic use, mother’s age, pre-pregnancy BMI, and gestational diabetes.

#### Gut microbiota of pregnant women

**Fig. 1:**
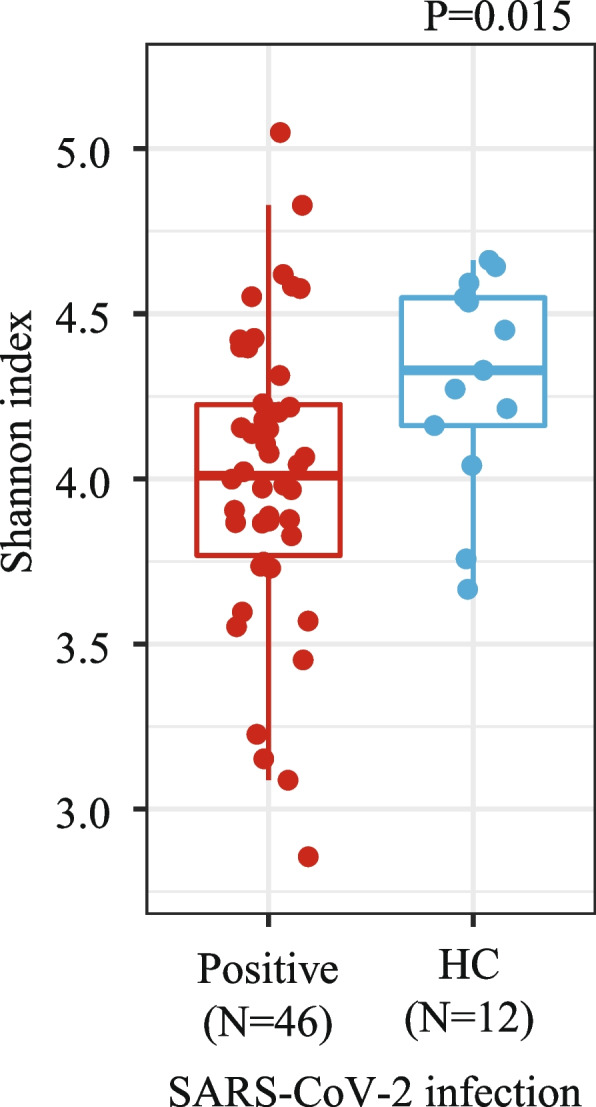
Gut microbial diversity of pregnant women differs by SARS-CoV-2 infection. Gut microbiota alpha diversity at the sequence variant (SV) level, of pregnant women with a positive SARS-CoV-2 diagnosis during pregnancy (Positive) or healthy controls (HC) was estimated using the Shannon index. *P* values were calculated by applying a linear regression model with Shannon diversity indexes as the dependent variable and SARS-CoV-2 infection (and other covariates, see methods) as the independent variables

Out of the 88 pregnant women included in the study, we obtained stool samples from 46 participants with positive SARS-CoV-2 diagnoses and 12 healthy participants (Table S3). Alpha diversity analyses showed that being diagnosed with SARS-CoV-2 during pregnancy was associated with lower gut microbial diversity compared to their healthy counterparts (Fig. 1, Table S4, Shannon index, Linear regression, *P* = 0.015). Gut microbial diversity did not differ by the timing of SARS-CoV-2 infection during pregnancy (Table S5, Shannon index, Linear Regression-Pairwise Estimated Marginal Means, *P*adj > 0.050). There were no statistically significant differences on the microbial compositional or microbial inter-person variability (beta diversity) by SARS-CoV-2 infection (Table S6, PERMANOVA and Betadisper test, *P* > 0.079).

We observed that gestational diabetes was significantly associated with higher gut microbial diversity (Table S4 and S5, Shannon, Linear regression, *P* < 0.001), and the gut microbiota composition varied by pre-pregnant BMI in this cohort (Sørensen index, PERMANOVA, *R*^2^ = 2.6%, *P* = 0.014).

We applied RFC to select the variables, including taxa at SV level and demographic variables, that predict SARS-CoV-2 infection and then we used the LIME algorithm to set abundance threshold values that best separate the two outcome groups. We found that SARS-CoV-2 infections were predicted (F1-score: 0.93) by a higher abundance of *Dialister* and lower of *Phascolarctobacterium faecium*, *Anaerostipes*, *Prevotella buccalis, Porphyromonas uenonis*, and *Bacteroides* (see each SV relative abundance threshold in Figure S2A).

In sum, SARS-CoV-2 infection during pregnancy was associated with reduced gut microbial diversity at delivery, regardless of the timing of the diagnosis. Finally, although no significant differences were observed for microbial composition, several taxa were found to be predictive of SARS-CoV-2 infection.

### Vaginal microbiota of pregnant women

**Fig. 2:**
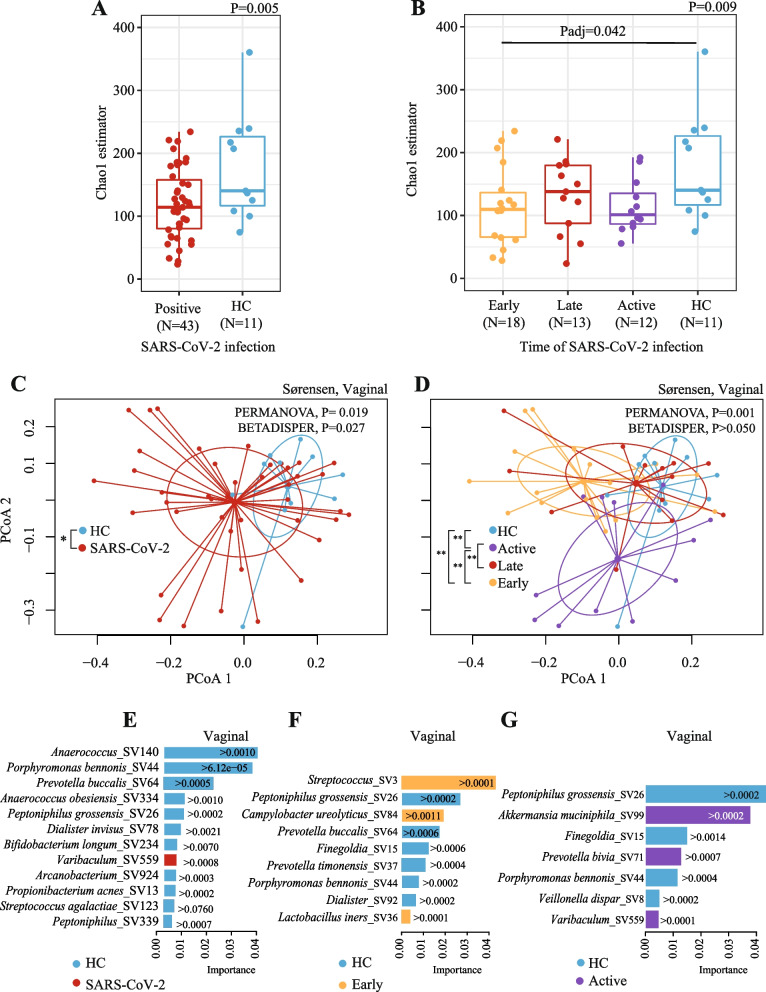
Vaginal microbiota of pregnant women differs by SARS-CoV-2 infection. **A** Alpha diversity is shown using the Chao1 estimator comparing pregnant women with SARS-CoV-2 infection during pregnancy and pregnant healthy controls (HC). **B** Alpha diversity is shown using the Chao1 estimator comparing pregnant women with SARS-CoV-2 infection early, late, or active vs. HC. Significance was determined using Linear regression and pairwise comparison with estimated marginal means. **C**,** D** Beta diversity analyses by groups: **C** all pregnant women with SARS-CoV-2 infection during pregnancy compared to HC; **D** pregnant women with SARS-CoV-2 infection early, late, or active compared to HC. Beta diversity comparisons were performed using PERMANOVA analysis with pairwise comparisons and BETADISPER for dispersion analysis. For PERMANOVA and BETADISPER analyses we used Sørensen dissimilarities. *P* values were all adjusted by false discovery rate. **E, F, G** Bacterial taxa (at the sequence variant or SV) were selected by the random forest classification (RFC) and ranked according to their importance in the classification. RFC comparisons are shown in **E** pregnant women with SARS-CoV-2 infection during pregnancy vs. HC, **F** pregnant women with early SARS-CoV-2 infection vs*.* HC, **G** pregnant women with active SARS-CoV-2 infection vs. HC. Bars’ colors indicate the comparison group (i.e., SARS-CoV-2 or HC), and each bar indicates the importance by which the increase on an SV predicts a particular comparison group. The selection of the variables for RFC was performed with Boruta algorithm. We also used the Local Interpretable Model-agnostic Explanation (LIME) to estimate a threshold of the abundance of the SV selected with Boruta that predicts a particular comparison group. *Padj < 0.050, **Padj < 0.010

A total of 54 vaginal samples were included in the microbiota analyses. We obtained 43 samples from pregnant women with SARS-CoV-2 infection and 11 samples from healthy controls (Table S3). We observed that pregnant women with SARS-CoV-2 infection exhibited lower vaginal microbial richness (Fig. 2A, Chao1 estimator, Linear regression, *P* = 0.005) compared to healthy controls. Moreover, pregnant women with early SARS-CoV-2 infection exhibited the lowest richness among all the pregnant women with SARS-CoV-2 infection when compared to healthy controls (Fig. 2B, Linear Regression-Pairwise Estimated Marginal Means, *P*adj = 0.042).

We also observed that pregnant women with SARS-CoV-2 infection exhibited a microbiota composition distinct from healthy controls, particularly those with early or active infections, clustering the furthest from that group (Fig. 2C, D, Sørensen index, PERMANOVA, *R*^2^ = 2.6%, and 8.6%, respectively *P* < 0.050). In addition, we observed that participants with SARS-CoV-2 infection showed higher inter-person vaginal microbiota variability than healthy controls who overall exhibited a more similar microbiota composition among individuals (Fig. 2C, D, Table S6, Sørensen index, BETADISPER analysis, adjusted for sample size differences, *P* = 0.027). Of note, differences in microbiota composition were detected only when evaluated with the Sørensen index (unweighted measurement), but not with Bray–Curtis dissimilarity (weighted measurement), indicating that the compositional changes are mainly driven by rare microbial taxa and not the highly abundant lactobacilli species.

As expected, the administration of antibiotics during delivery showed a significant association with the vaginal microbiota diversity in this cohort (Sørensen index, PERMANOVA, *R*^2^ = 6.0%, *P* = 0.030, Table S6). However, antibiotic use was similarly distributed among SARS-CoV-2-infected and healthy participants; thus, the outcome of SARS-CoV-2 on the vaginal microbiota is relevant despite antibiotic use (Table 1, and Table S1).

We applied RFC and LIME including taxa at SV level and demographic variables. We found that SARS-CoV-2 infections were predicted (F1-score: 0.94) a higher abundance of *Varibaculum* and by a lower abundance of *Anaerococcus, Porphyromonas bennonis*, *Prevotella buccalis, Anaerococcus obesiensis, Peptoniphilus obesiensis, Dialister invisus, Bifidobacterium longum, Arcanobacterium, Propionibacterium acnes, and Streptococcus agalactiae* and *Peptoniphilus* in the vaginal samples (Fig. 2E, Figure S1A).

Furthermore, we investigated whether the timing of SARS-CoV-2 infection impacted the vaginal microbiota composition. We found that pregnant women with SARS-CoV-2 infection in early pregnancy were predicted (F1-score: 0.91) by a higher abundance of *Streptococcus, Campylobacter ureolyticus*, and *Lactobacillus iners* and by a lower abundance of *Peptoniphilus grossensis, Prevotella buccalis, Finegoldia, Prevotella timonensis, Porphyromonas bennonis*, and *Dialister* (Fig. 2F, Figure S1B). Pregnant women with active SARS-CoV-2 infection were predicted (F1-score: 0.83) by a higher abundance of *Akkermansia muciniphila*, *Prevotella bivia*, and *Varibaculum* and by a lower abundance of *Peptoniphilus grossensis*, *Finegoldia*, *Porphyromonas bennonis*, and *Veillonella dispar* (Fig. 2G, Figure S1C). No SVs was predictive of pregnant women with late SARS-CoV-2 infection.

Additionally, we classified the vaginal microbiota into community state types (CSTs) as previously done [50]. Each CST is characterized by the dominance of a specific specie of *Lactobacillus* (i.e., *L. crispatus* (CST-I)*, L. gasseri* (CST-II)*, L. iners* (CST-III)*, L. jensenii* (CST-V)) or the absence of *Lactobacillus* dominance (CST-IV) [50]. Most of the pregnant women recruited for this study were classified on the CST-I (42.2%) followed by CST-III (37.5%). We did not find significant differences in CSTs distribution by SARS-CoV-2 infection or time of diagnosis compared to their healthy counterpart (Table S7, Fisher’s exact test, *P* > 0.050).

### Oral microbiota of pregnant women

**Fig. 3:**
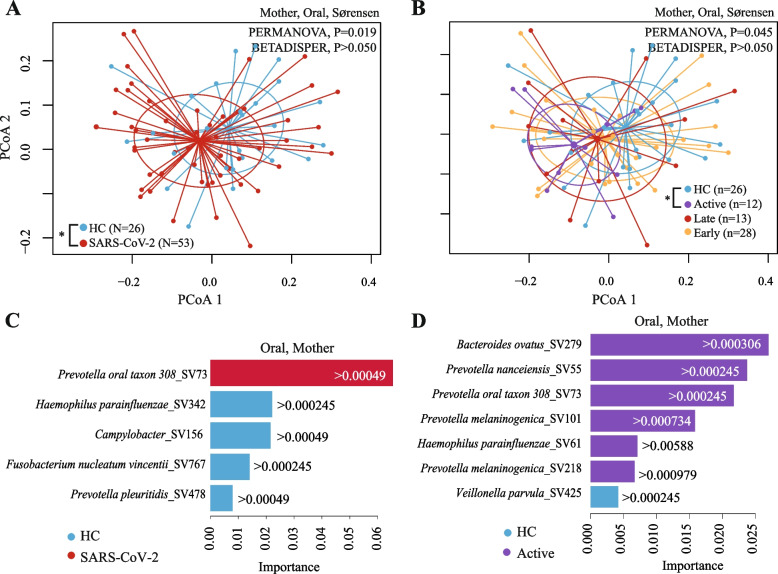
Oral microbiota of pregnant women differs by SARS-CoV-2 infection. Beta diversity analyses by groups: **A** pregnant women with SARS-CoV-2 infection compared to HC or **B** pregnant women with early, late, or active SARS-CoV-2 infections compared to HC. Beta diversity comparisons were performed using PERMANOVA analysis with pairwise comparisons and BETADISPER for dispersion analysis. For PERMANOVA and BETADISPER analyses, we used Sørensen dissimilarities. *P* values were all adjusted by false discovery rate. **C**, **D** Bacterial taxa (at the sequence variant or SV) were selected by the random forest classification (RFC) and ranked according to their importance in the classification. RFC comparisons are shown in **C** pregnant women with SARS-CoV-2 infection compared to HC; **D** pregnant women with active SARS-CoV-2 infection compared to HC. Bar colors indicate the comparison group (i.e., SARS-CoV-2 or HC); and each bar indicates the importance by which the increase in an SV predicts a particular comparison group. The selection of the variables for RFC was performed using the Boruta algorithm. We also used the Local Interpretable Model-agnostic Explanation (LIME) to estimate a threshold of the abundance of the SV selected with Boruta that predicts a particular comparison group. *Padj < 0.050, **Padj < 0.010

Out of the 78 pregnant women providing oral samples, 53 were positive for SARS-CoV-2 during pregnancy and 25 were healthy controls (Table S3). There were no alpha diversity differences between the groups (Table S4, S5, Shannon index or Chao1 estimator, Linear regression, *P* > 0.050). However, the oral microbiota composition was better explained by race (PERMANOVA, *R*^2^ = 4.7%, *P* = 0.023), followed by SARS-CoV-2 infection (Fig. 3A. PERMANOVA, *R*^2^ = 4.5%, *P* = 0.019). Particularly, pregnant women with active SARS-CoV-2 infection have significantly different oral microbiota compared to healthy controls (Fig. 3B, Sørensen index, Pairwise PERMANOVA, *P*adj = 0.042).

RFC and LIME algorithms showed that the oral microbiota at the SV level of pregnant women with SARS-CoV-2 infection were predicted (F1-score: 0.92) by a higher abundance of *Prevotella* oral taxon 308 and lower abundance of *Haemophilus parainfluenzae*, *Campylobacter*, *Fusobacterium nucleatum vicentii,* and *Prevotella pleuritidis* (Fig. 3C, Figure S1D). Active SARS-CoV-2 infection were predicted (F1-score = 0.87) by a higher abundance of SVs belonging to *Bacteroides ovatus, Prevotella nanceiensis*, *Prevotella* oral taxon 308, *Prevotella melaninogenica*, and *H. parainfluenzae* and by a lower abundance of *Veillonella parvula* (Fig. 3D, Figure S1F).

Altogether, the oral microbiota composition of pregnant women was remarkably different for pregnant women with SARS-CoV-2 infection compared to healthy controls.

### SARS-CoV-2 infection during pregnancy is associated with alterations in the microbiota of the offspring

#### Stool microbiota of infants

We obtained the infant’s stool samples 1–2 days post-partum (meconium). From the total number of infants, we could collect and analyze 38 samples from infants born to mothers with SARS-CoV-2 infection during pregnancy and 10 samples from infants born to healthy controls (Table S3). Contrary to expectations, SARS-CoV-2 infection during pregnancy did not associate with alpha or beta diversity of the offspring gut microbiota (Table S4, S5, and S6). However, pre-pregnancy BMI was negatively associated with alpha diversity (Table S4 and S5, Shannon index, Linear regression, *P* = 0.032) and explained most of the microbial composition of the infant’s gut (PERMANOVA, Bray–Curtis dissimilarity, *R*^2^ = 4.0%, *P* = 0.025) together with infant weight at birth (PERMANOVA, Bray–Curtis dissimilarity and Sørensen index, *R*^2^ = 3.9%, *P* < 0.020). As expected, the gut microbiota composition varied by delivery mode (PERMANOVA, Bray–Curtis dissimilarity, *R*^2^ = 4.7%, *P* = 0.010).

The overall gut microbiota composition was not significantly associated with SARS-CoV-2 infection in infants. Yet, the abundance of specific bacterial taxa could distinguish between infants born to pregnant women with SARS-CoV-2 and those born to healthy mothers. Particularly, RFC and LIME algorithms showed that gut SVs of infants from pregnant women with SARS-CoV-2 infections were predicted (F1-score: 0.98) by a higher abundance of *Enterococcus* and by lower abundance of H. *parainfluenzae* (Figure S2B). RFC was performed including delivery mode and antibiotic as variables to be selected in addition to taxa.

Furthermore, we measured levels of fecal calprotectin in the infants’ stool. Fecal calprotectin is a non-invasive biomarker that robustly correlates with gut inflammation [51–56]. Moreover, fecal calprotectin has been shown to be elevated in infants born to pregnant women with chronic inflammation [47]. We observed a marginal, but not significant, difference in fecal calprotectin levels between mothers with and without SARS-CoV-2 infection (Kruskal–Wallis analysis, *P* = 0.052). However, infants born to pregnant women with active SARS-CoV-2 infection exhibited higher fecal calprotectin levels compared to healthy controls (Figure S4A, pairwise Kruskal–Wallis, *P*adj = 0.045). We also explored associations of fecal calprotectin levels and taxa abundance. We found that the abundance of several taxa in active SARS-CoV-2 infections were positively associated with fecal calprotectin, but the correlations were not statistically significant after the corrections (Figure S4B). On the healthy control group, abundance of genus *Leptothrix* from the Betaproteobacteria class was significantly positively associated with levels of fecal calprotectin (Figure S4B, Spearman correlation test, *P*adj = 0.007).

#### Oral microbiota of infants

A total of 47 oral samples were obtained from infants after 1–2 days post-partum: 29 from infants born to SARS-CoV-2-infected pregnant women and 18 born to healthy controls. Oral microbiota alpha diversity was not statistically significant between infants based on their mothers’ SARS-CoV-2 diagnosis (Table S4, S5, Shannon index and Chao1 estimator, Linear regression, *P* > 0.050).

**Fig. 4:**
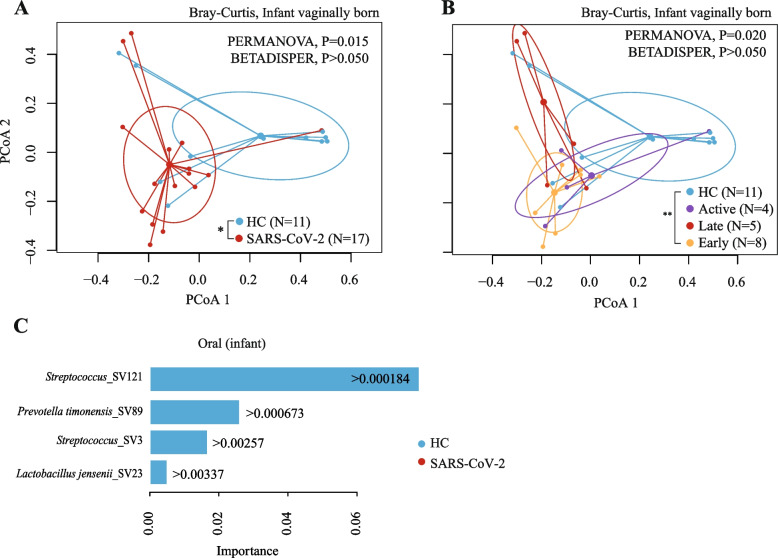
The oral microbiota of infants born to pregnant women with SARS-CoV-2 is altered. Beta diversity analyses by groups: **A** infants born to pregnant women infected with SARS-CoV-2 during pregnancy compared to infants born to pregnant healthy controls (HC); **B** infants born to pregnant women infected with SARS-CoV-2 early or late during pregnancy or with active infection, compared to HC. Beta diversity comparisons were performed using PERMANOVA analysis with pairwise comparisons and BETADISPER for dispersion analysis. For PERMANOVA and BETADISPER analyses, we used Sørensen dissimilarities. *P* values were all adjusted by false discovery rate. **C** Bacterial taxa (at the amplicon sequence variant or SV) were selected by the random forest classification (RFC) and ranked according to their importance in the classification for infants born to pregnant women with SARS-CoV-2 compared to HC. Bar colors indicate the comparison group (i.e., SARS-CoV-2 or HC); and each bar indicates the importance by which the increase in an SV predicts a particular comparison group. The Boruta algorithm was used to select variables for RFC. We also used the Local Interpretable Model-agnostic Explanation (LIME) to estimate a threshold of the abundance of the SV selected with Boruta that predicts a particular comparison group. *Padj < 0.050, **Padj < 0.010

However, infants born to pregnant women with SARS-CoV-2 infection exhibited a significantly different bacterial composition compared to infants born to healthy controls (PERMANOVA, *R*^2^ = 7.9%, *P* = 0.013). Particularly, infants born to pregnant women with active SARS-CoV-2 infection clustered the furthest from those born to healthy controls (Table S6, pairwise PERMANOVA, *R*^2^ = 11.6%, *P*adj = 0.002). As expected, although to a lesser degree, the delivery mode also associated with the oral microbiota composition (PERMANOVA, *R*^2^ = 4.6%, *P* = 0.010). We then stratified infants by mode of delivery to eliminate its effect. Here, we observed that infants born vaginally to mothers with SARS-CoV-2 infection (*N* = 17) exhibited a significantly different microbial composition than those born to healthy controls (*N* = 11, Fig. 4A, Table S6, Bray–Curtis dissimilarity, PERMANOVA, *P* < 0.015). Furthermore, infants born vaginally to pregnant women with early infection presented an oral microbiota that separated the furthest from those infants born vaginally to healthy controls (Fig. 4B, Table S6, Bray–Curtis dissimilarity, PERMANOVA, *P* < 0.015). No variable significantly explained composition in cesarean-born infants. Interestingly, oral infant microbial composition differed only when assessed by Bray–Curtis dissimilarity, indicating the differences observed were due to changes in highly abundant taxa (Table S3).

RFC and LIME algorithms run for microbial taxa at the SV level also including demographic variables showed that infants born to mothers with SARS-CoV-2 infection during pregnancy were predicted (F1-score: 0.99) by a low abundance of *Streptococcus* (two different SVs)*, Prevotella timonensis*, and *Lactobacillus jensenii* (Fig. 4C).

### Pregnant women with early and late but not active SARS-CoV-2 infections transferred viral antibodies to their infants

Finally, we measured immunoglobulin G (IgG) antibody levels against nucleocapsid protein (NP) in cord blood to determine whether maternal antibodies were vertically transferred to their infants. We found significantly higher IgG levels in cord blood from pregnant women with early and late infections, but not with active infection, compared to healthy controls (Figure S3).

## Discussion

We observed that SARS-CoV-2 infection during pregnancy, particularly in early pregnancy or active infection at the time of delivery, was associated with perturbations of the vaginal, gut, and oral microbiota of pregnant women compared to healthy controls. Moreover, the microbiome alterations in pregnant women were reflected in the infant’s oral microbiome. To our knowledge, this is the first study exploring the impact of SARS-CoV-2 infection on the vaginal and oral microbiota of pregnant women and their effect on the offspring’s gut and oral microbiota.

First, we observed a decrease in gut microbial diversity in infected pregnant women. Low gut microbial diversity has been linked to negative health outcomes [57, 58]. The fact that these changes were observed even if the infection was early in pregnancy suggests the long-lasting effect of SARS-CoV-2 infection on gut microbial diversity [30]. Our findings are similar to another study on non-pregnant Chinese individuals, in which COVID-19 was associated with reduced gut microbial richness [59] even lower than in H1N1 hospitalized patients [60], and patients exhibited a long-lasting effect on the microbiota composition for at least 6 months post-infection [59]. Yet, a recent study including pregnant women from Mexico whose stool samples were positive for SARS-CoV-2, despite them being asymptomatic or negative on nasopharyngeal swabs (suggesting earlier infection) found no statistical differences in gut microbiota alpha diversity but showed differences in the gut microbiota composition and in specific taxa [30]. Differences in results might be due to the presence of SARS-CoV-2 in the gut—we did not assess SARS-CoV-2 in stools, to geographical differences in the microbiota that could result in a varying response to the viral infection, or to differences in the circulating viral strain.

A recent study from Spain reported that the overall composition of the nasopharyngeal microbiota differs in pregnant women with SARS-CoV-2 infection compared to healthy controls [29]. Specifically, the authors observed a higher abundance of *Prevotellaceae* family (*Bacteroidales* order) in pregnant women with active SARS-CoV-2 infection. A higher abundance of members of the *Prevotellaceae* family in the oral cavity predicted SARS-CoV-2 infection in the pregnant cohort included in this study. Members of this bacteria family, such as *P. intermedia*, are considered the main bacterial species implicated in periodontitis [61]. Similarly, *H. parainfluenzae*, an oral commensal associated with beneficial immunomodulatory effects, is decreased in the pregnant women with SARS-CoV-2 included in this study [62]. Conversely, *F. nucleatum,* decreased in pregnant women with SARS-CoV-2 infection in our study, is one of the most prevalent species and by far the most prevalent oral species implicated in adverse pregnancy outcomes [63, 64]. Overall, our results suggest that SARS-CoV-2 infection plays an important role in dictating the abundance of bacteria linked to immune regulation and pregnancy outcomes.

As for the vaginal microbiota, different studies have reported a stable vaginal microbiota composition even during pregnancy, particularly in the Caucasian population [65, 66], which includes most of the participants of this study. We demonstrate that SARS-CoV-2 infection impacts the vaginal microbiota richness and composition. Compositional vaginal microbiota changes observed in pregnant women with active SARS-CoV-2 infection may be the consequence of alterations in the vaginal epithelial environment [67] and interactions with the immune system. Although viral particles of SARS-CoV-2 have not been detected in the vaginal fluid [68–70], this pulmonary infection promotes strong systemic inflammatory responses [71]. This pro-inflammatory immune tone on the epithelia, including the vagina, may limit or favor the survival of certain taxa. Furthermore, we observed a higher vaginal microbial heterogeneity of low abundance taxa among infected women compared to controls. This high heterogeneity was previously observed in a metagenomic study with post-menopausal women [68], where not only the microbial diversity but also the proportion of bacterial transcript varied considerably among SARS-CoV-2-infected participants [68]. Such inter-individual variation may be due to the differences in the personal physiological response to SARS-CoV-2 disease severity, the individual’s hormonal profile (e.g., early, or late in pregnancy), and/or health status. Since low vaginal microbial richness and diversity are frequently associated with a healthy state, opposite to the high richness found in bacterial vaginosis and in other inflammatory states [72], we expected to find higher richness in infected women. However, mothers with SARS-CoV-2 infection exhibited lower vaginal microbial richness (although no difference for microbial diversity, Shannon index) compared to healthy controls, with a decrease of several bacterial taxa associated with bacterial vaginosis. The clinical implications of this decreased richness in the vaginal microbiota of SARS-CoV-2 pregnant women deserve further investigation.

Moreover, differences on the pregnant women’s vaginal microbiota by SARS-CoV-2 infections were reflected in the offspring. Vaginally delivered infants have different representation of bacterial species expected to be the first colonizers of the infant’s oral cavity [73], dependent on their mother’s SARS-CoV-2 status. The composition of the oral microbiome is established early in life, is stable throughout life [74, 75], and it has implications for long-term health [76, 77]. Despite oral microbiota changes in infants, we did not observe an association of SARS-CoV-2 infection with the offspring’s gut microbiota diversity; although we observed that the abundance of some taxa could predict the infant gut microbiota by mothers’ SARS-CoV-2 status. Similarly, others have reported no changes in overall gut bacterial diversity in infants born to mothers with SARS-CoV-2 infection but differences in specific taxa [78]. This suggest that other maternal microbiotas may have a larger contribution to the offspring gut microbiota than the mother’s vaginal microbiota. In fact, almost 60% of the infant gut microbiota composition can be traced to different maternal microbial sources including not only the vaginal microbiota but also breast milk, skin, saliva, and feces [7].

Our results indicate that infants born to pregnant women with SARS-CoV-2 active infection have higher levels of fecal calprotectin, indicative of an early gut inflammation. Elevated fecal calprotectin has been observed in infants born to pregnant women with inflammatory bowel diseases [47]. Similarly, others have found that infants born to mothers with active SARS-CoV-2 infection exhibit induction of T-cell-associated cytokines (IL33, NFATC3, and CCL21) compared to infants born to healthy mothers [79]. Therefore, infants born to mothers with active SARS-CoV-2 infections may present a pro-inflammatory immune tone early in life.

Finally, we found that pregnant women with active SARS-CoV-2 show a lower, although not significant, prevalence of vaginal delivery (42.9 vs 61.5%). The literature, thus far, is inconsistent on estimating the risk for C-section on pregnant women with SARS-CoV-2 [27, 80], yet larger studies are needed to fully determine this risk.

Study limitations include a small sample size, particularly for infants where stratification by mode of delivery greatly reduced the power to discriminate between SARS-CoV-2 infection and healthy control groups. Additionally, participants were mostly non-Hispanic Whites, followed by Hispanics, which limits the conclusions to this population sector, particularly important for vaginal microbiota which is highly dependent on the racio-ethnic background [50]. Additionally, the use of antibiotics during delivery was present in a significant portion of the cohort included in the study (37%). The use of antibiotics was mostly due to treatment of group B streptococcus of infected mothers, and although it was similarly distributed among groups, the effect on non-antibiotic users would be important to investigate. Another limitation was the availability of SARS-CoV2 diagnostics during the first year of the COVID-19 pandemic when our study was conducted, which may have led to underreporting of infections during pregnancy. However, our antibody results, using cord blood as a proxy for the presence of maternal antibodies to NP IgG aided proper classification of our study participants.

We also had limited racial diversity of controls, as some controls were recruited pre-pandemic for another study. Therefore, reporting on both demographics and perinatal outcomes has limited relevance in this manuscript, as controls were chosen out of convenience of the timing of delivery and sample collection, often a scheduled cesarean delivery, unlike cases who were approached when identified as having had prior SARS-CoV-2 infection and presenting to the labor floor. Despite these limitations, our study conveys differences in the microbiota of the birthing parent, implication of which will need to be explored in future research studies.

## Conclusions

Our study highlights microbial changes associated with SARS-CoV-2 infection, providing a baseline to understand the potential clinical implications beyond immediate infection risks. As alterations in the microbiota can have health implications in the infant, these changes are important to characterize, and potential treatment can be explored to counter these changes. For example, besides avoiding SARS-CoV-2 infections, actions such as probiotic intake and a diet focused on microbiota balance might be a benefit for the already infected patients. Emphasis might be on parental intervention, since they will be the principal source of microbiota colonization of the infant. As COVID-19 becomes more endemic and as the COVID-19 vaccine is widely recommended in pregnancy, it will be important to address all risks to SARS-CoV-2 infection, which with future research may include changes in the gut microbiota and possible therapies to prevent such changes.

## Supplementary Information

**Additional file 1.** Supplementary figures (Figures S1 to S4) and tables (Tables S1 to S7).

## Data Availability

Sequencing and de-identified clinical data were deposited in the NCBI database, BioProject ID: PRJNA871082.

## Abbreviations

BMI: Body mass index
CST: Community state type
HC: Healthy control
IgG: Immunoglobulin G
LIME: Local Interpretable Model-agnostic Explanation
NP: Nucleocapsid protein
SV: Sequence variant
OD: Optical density
PERMANOVA: Permutational multivariate analysis of variance
RFC: Random forest classification
