# Supplementary material for: The microbiota of pregnant women with SARS-CoV-2 and their infants
Source: Microbiome. 2023 Jun 26;11:141. doi: 10.1186/s40168-023-01577-z (PMC10291758; doi:10.1186/s40168-023-01577-z)
Supplement: Supplementary file 2 — Additional file 1. Supplementary figures (Figures S1 to S4) and tables (Tables S1 to S7). [file 40168_2023_1577_MOESM1_ESM.zip › 40168_2023_1577_MOESM1_ESM_AMC.docx]

**SUPPLEMENTARY FIGURES**

**
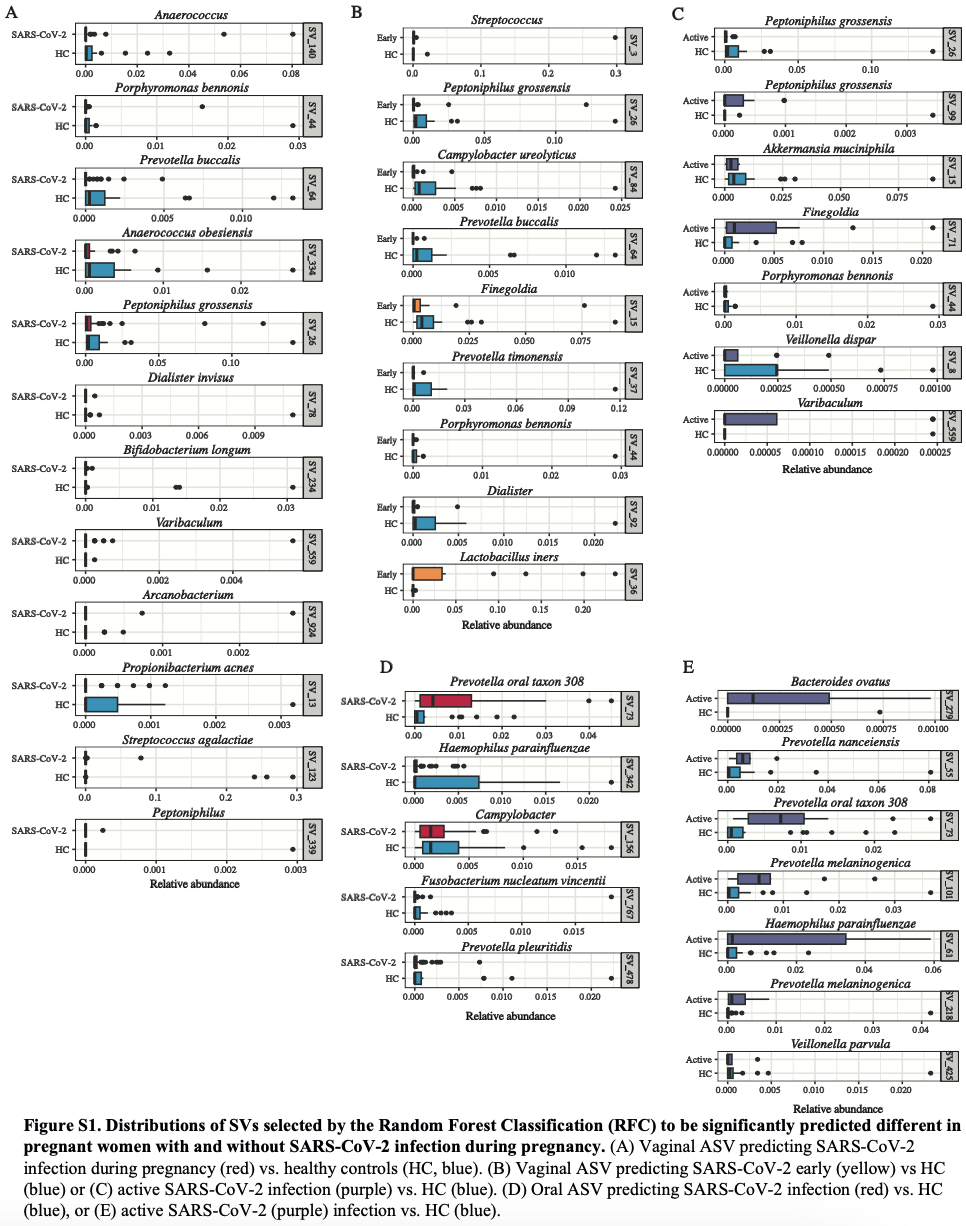
**

**
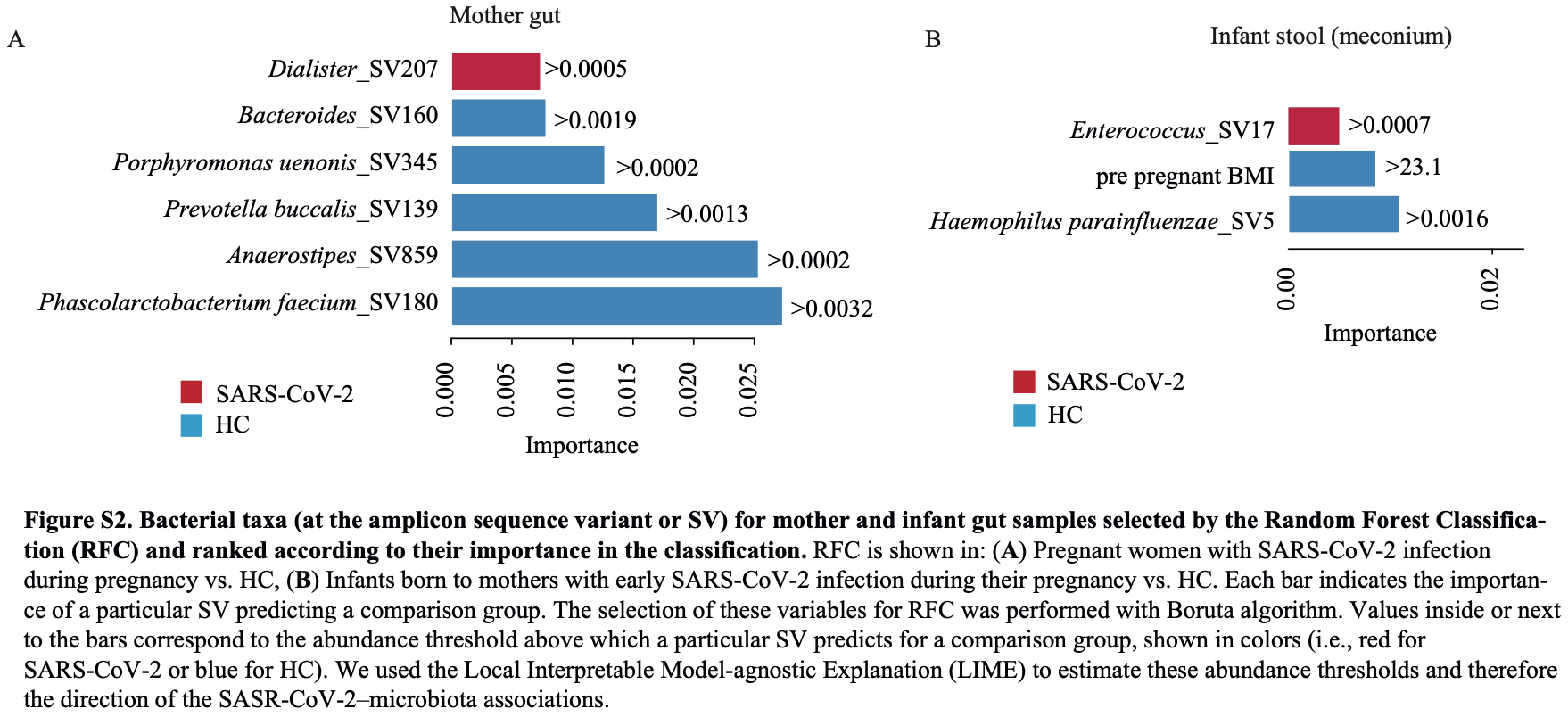
**

**
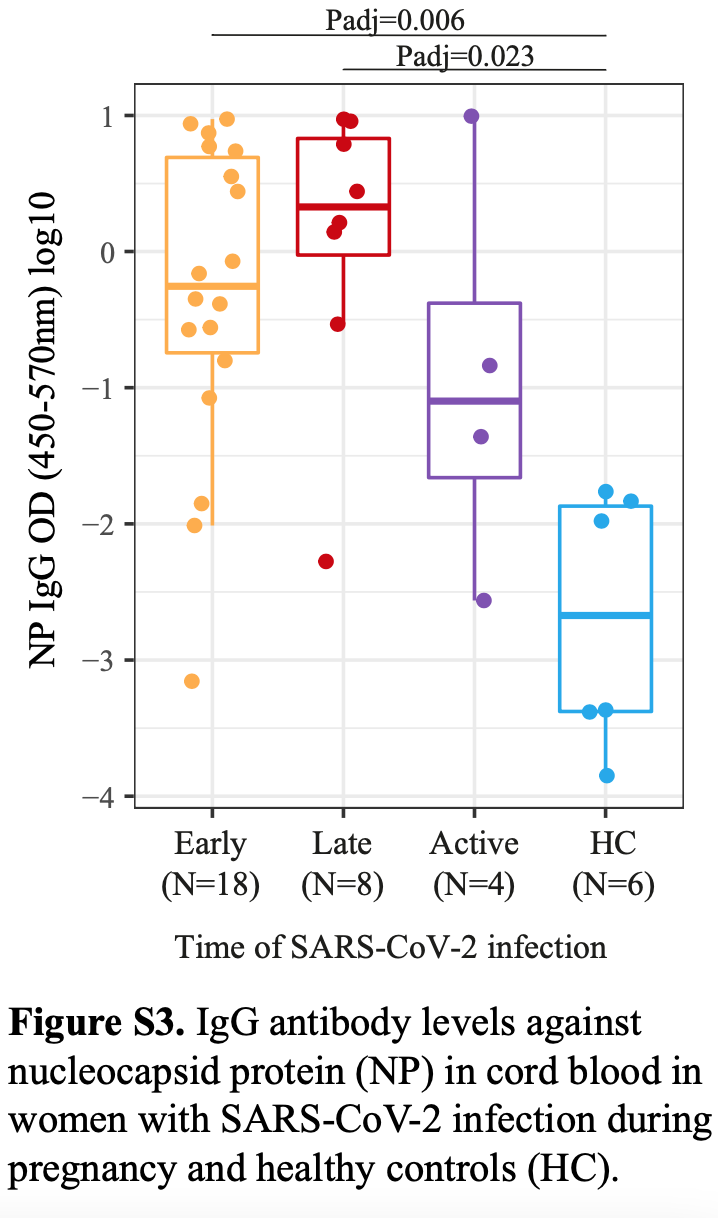
**


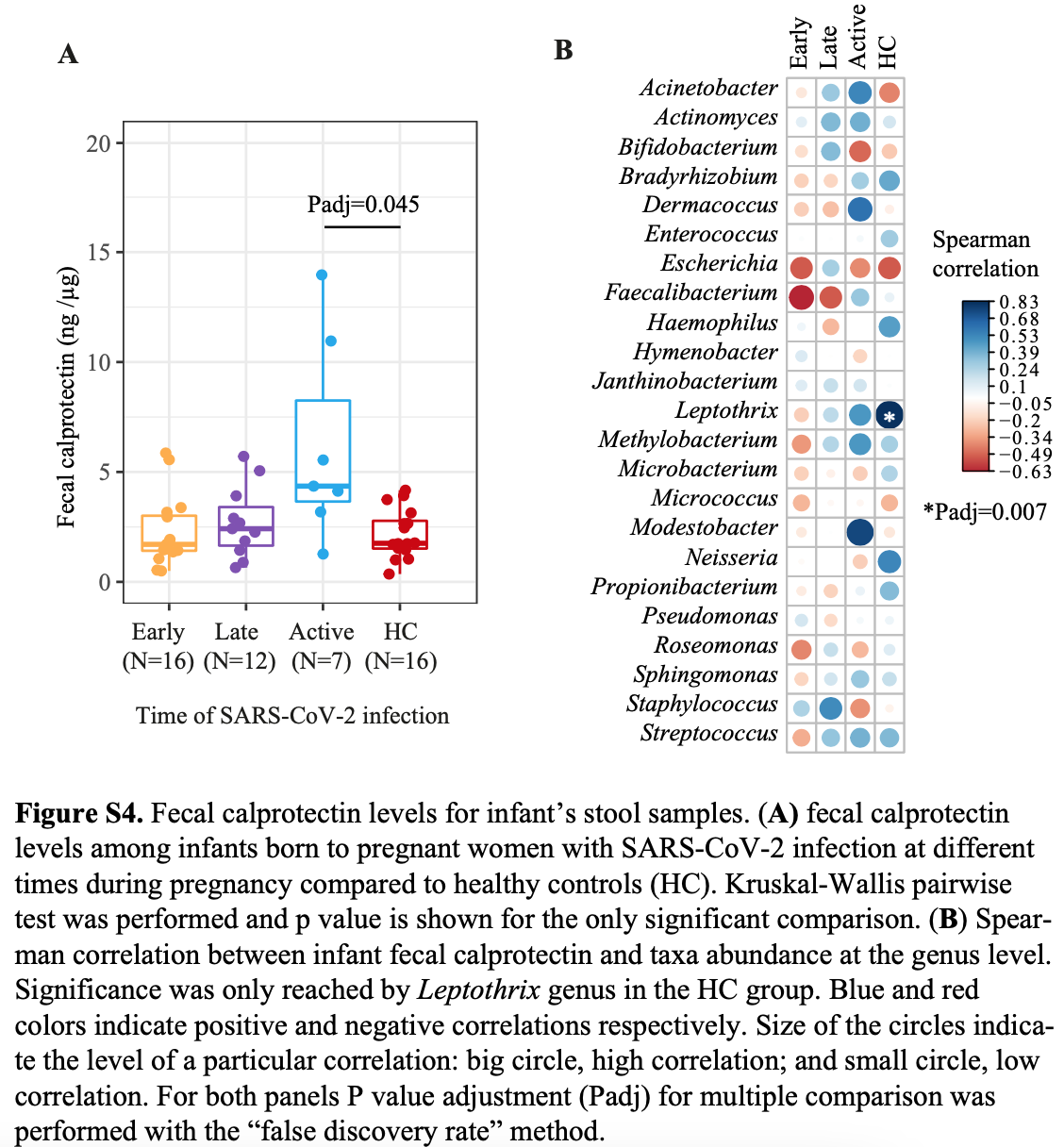


**SUPPLEMENTARY TABLES**

| **Table S1**. Demographic and clinical variables for women infected with SARS-CoV-2 during early or late in pregnancy or having active infections at delivery and healthy controls. | | | | | | |
| --- | --- | --- | --- | --- | --- | --- |
| **Demographics and clinical variables** | **SARS-CoV-2 positive pregnant women (N=62)** | | | **Healthy controls pregnant women (N=26)** |  |  |
|  | **Early infection (N=31)** | **Late infection (N=17)** | **Active infection (N=14)** |  | **TOTAL (N=88)** | ***P value &*** |
| ***Age*** |  |  |  |  |  | 0.037 |
| Mean (SD) | 31.0 (7.19) | 30.1 (4.65) | 32.1 (5.90) | 33.4 (4.96) | 31.7 (5.97) |  |
| ***Body mass index*** |  |  |  |  |  |  |
| **Pre-pregnancy** |  |  |  |  |  | 0.789 |
| Mean (SD) | 30.7 (6.49) | 32.4 (8.01) | 30.9 (6.85) | 29.5 (7.33) | 30.7 (7.05) |  |
| **Pre-pregnancy category** |  |  |  |  |  | 0.361 |
| Underweight | 1 (3.2%) | 0 (0%) | 0 (0%) | 1 (3.8%) | 2 (2.3%) |  |
| Normal | 4 (12.9%) | 5 (29.4%) | 2 (14.3%) | 4 (15.4%) | 15 (17.0%) |  |
| Overweight | 11 (35.5%) | 3 (17.6%) | 6 (42.9%) | 13 (50.0%) | 33 (37.5%) |  |
| Obese | 15 (48.4%) | 9 (52.9%) | 6 (42.9%) | 8 (30.8%) | 38 (43.2%) |  |
| ***Race*** |  |  |  |  |  | 0.145 |
| Non-Hispanic White | 14 (45.2%) | 6 (35.3%) | 9 (64.3%) | 18 (69.2%) | 47 (53.4%) |  |
| Hispanic or Latino | 13 (41.9%) | 8 (47.1%) | 3 (21.4%) | 2 (7.7%) | 26 (29.5%) |  |
| Non-Hispanic Black | 4 (12.9%) | 3 (17.6%) | 0 (0%) | 4 (15.4%) | 11 (12.5%) |  |
| Non-Hispanic Asian | 0 (0%) | 0 (0%) | 2 (14.3%) | 2 (7.7%) | 4 (4.5%) |  |
| ***SARS-CoV-2 comorbidities*** |  |  |  |  |  |  |
| **Type 2 diabetes** | 3 (9.7%) | 0 (0%) | 1 (7.1%) | 2 (7.7%) | 6 (6.8%) | 0.316 |
| **Cardiovascular disease** | 6 (19.4%) | 3 (17.6%) | 3 (21.4%) | 4 (15.4%) | 16 (18.2%) | 0.197 |
| ***Pregnancy outcomes*** |  |  |  |  |  |  |
| **Vaginal delivery** | 23 (74.2%) | 14 (82.4%) | 6 (42.9%) | 16 (61.5%) | 59 (67.0%) | 0.093 |
| **Preeclampsia** | 5 (16.1%) | 1 (5.9%) | 3 (21.4%) | 2 (7.7%) | 11 (12.5%) | 0.167 |
| Missing | 1 (3.2%) | 0 (0%) | 0 (0%) | 0 (0%) | 1 (1.1%) |  |
| Preterm (<37 weeks) | 6 (19.4%) | 4 (23.5%) | 4 (28.6%) | 3 (11.5%) | 17 (19.3%) | 0.516 |
| **Gestational diabetes** | 5 (16.1%) | 4 (23.5%) | 4 (28.6%) | 3 (11.5%) | 16 (18.2%) | 0.279 |
| **Antibiotic during delivery** | 10 (32.3%) | 5 (29.4%) | 9 (64.3%) | 12 (46.2%) | 36 (40.9%) | 0.457 |
| **Antibiotic before delivery** | 3 (9.7%) | 1 (5.9%) | 1 (7.1%) | 0 (0%) | 5 (5.7%) | 0.382 |
| ***Vaccinated against SARS-CoV-2*** | 0 (0%) | 1 (5.9%) | 0 (0%) | 9 (34.6%) | 10 (11.4%) | 1.00E-04 |
| & Fisher's exact test for categorical variables and Kruskal-Wallis test for continuous variables | | | | |  |  |

| **Table S2.** Demographic and clinical variables for infants from women infected with SARS-CoV-2 during early or late in pregnancy or having active infections at delivery and healthy controls. | | | | | | |
| --- | --- | --- | --- | --- | --- | --- |
| **Demographics and clinical variables** | **Infants born to pregnant women with early infection (N=21)** | **Infants born to pregnant women with late infection (N=16)** | **Infants born to pregnant women with active infection (N=10)** | **Infants born to Healthy controls pregnant women (N=21)** | **TOTAL (N=68)** |  |
|  |  |  |  |  |  | ***P value&*** |
| ***Gender*** |  |  |  |  |  | 0.188 |
| Female (%) | 14 (66.7%) | 7 (43.8%) | 8 (80.0%) | 10 (47.6%) | 39 (57.4%) |  |
| ***Infant weight (g)*** |  |  |  |  |  | 0.5175 |
| Mean (SD) | 3040 (733) | 3400 (556) | 3180 (505) | 3420 (444) | 3270 (590) |  |
| ***Delivery mode*** |  |  |  |  |  | 0.178 |
| Vaginal | 15 (71.4%) | 13 (81.3%) | 4 (40.0%) | 14 (66.7%) | 46 (67.6%) |  |
| ***NICU admission*** | 6 (28.6%) | 1 (6.3%) | 1 (10.0%) | 2 (9.5%) | 10 (14.7%) | 0.196 |
| & Fisher's exact test | |  |  |  |  |  |

| **Table S3.** Number of samples per group by sampling body site for pregnant women and infants. | | | | | |
| --- | --- | --- | --- | --- | --- |
| **Body site** | **SARS-CoV-2-positive pregnant women with (N=62)** | | | **Healthy controls pregnant women (N=26)** | **TOTAL** |
|  | **Early infection (N=31)** | **Late infection (N=17)** | **Active infection (N=14)** |  |  |
| Gut (anal swab) | 18 | 14 | 14 | 12 | **58** |
| Tongue | 28 | 13 | 12 | 25 | **78** |
| Vagina | 18 | 13 | 12 | 11 | **54** |
|  | **Infants born to mothers:** | | | |  |
|  | **with SARS-CoV-2 early infection (N=21)** | **with SARS-CoV-2 late infection (N=16)** | **with active SARS-CoV-2 infection (N=10)** | **Healthy controls (N=21)** |  |
| Gut (meconium) | 17 | 13 | 8 | 10 | **48** |
| Tongue | 14 | 7 | 8 | 18 | **47** |
| *Diagnosis of SARS-CoV-2 positive at delivery | | |  |  |  |

| **Table S4.** Linear model estimates and P values per SARS-CoV-2 infections by sample type | | | | | | | | | | | | | | | | | | | |  |
| --- | --- | --- | --- | --- | --- | --- | --- | --- | --- | --- | --- | --- | --- | --- | --- | --- | --- | --- | --- | --- |
|  |  | | | | | |  | |  | |  | |  | |  | |  | |  | |
| *Independent variables:* | *Dependent variables:* | | | | | | | | | | | | | | | | | | | |
|  | **Mother gut** | | | | **Mother vagina** | | | | **Mother tongue** | | | | **Infant gut** | | | | **Infant tongue** | | | |
|  | Shannon | | Chao1 | | Shannon | | Chao1 | | Shannon | | Chao1 | | Shannon | | Chao1 | | Shannon | | Chao1 | |
| SARS-CoV-2 pos (estimates) | -0.32 | | -56 | |  | | -60.7 | |  | |  | |  | |  | |  | |  | |
| (P value) | 0.015 | | 0.085 | |  | | 0.006 | |  | |  | |  | |  | |  | |  | |
| Gestational diabetes (estimates) | 0.469 | | 82.78 | |  | |  | |  | |  | |  | | 49.04 | |  | |  | |
| (P value) | 5E-04 | | 0.012 | |  | |  | |  | |  | |  | | 0.064 | |  | |  | |
| Antibiotic during delivery (estimates) | 0.168 | |  | |  | |  | |  | |  | |  | |  | |  | |  | |
| (P value) | 0.112 | |  | |  | |  | |  | |  | |  | |  | |  | |  | |
| Pre pregnant BMI (estimates) |  | |  | | 0.026 | | 1.873 | |  | |  | | -0.05 | |  | |  | |  | |
| (P value) |  | |  | | 0.128 | | 0.128 | |  | |  | | 0.032 | |  | |  | |  | |
| Delivery mode Vaginal (estimates) | |  | |  | |  | |  | |  | | -0.72 | |  | |  | |  | |  |
| (P value) |  | |  | |  | |  | |  | |  | | 0.059 | |  | |  | |  | |
| Infant weight g (estimates) |  | |  | |  | |  | |  | |  | | 5E-04 | |  | |  | |  | |
| (P value) |  | |  | |  | |  | |  | |  | | 0.069 | |  | |  | |  | |
| Mother age (estimates) |  | |  | |  | |  | |  | |  | |  | |  | | 0.042 | |  | |
| (P value) |  | |  | |  | |  | |  | |  | |  | |  | | 0.111 | |  | |
| Race Black (estimates) |  | |  | |  | |  | |  | |  | |  | |  | |  | | -9.48 | |
| (P value) |  | |  | |  | |  | |  | |  | |  | |  | |  | | 0.892 | |
| Race Other (Hispanics) (estimates) | |  | |  | |  | |  | |  | |  | |  | |  | | -21.7 | |  |
| (P value) |  | |  | |  | |  | |  | |  | |  | |  | |  | | 0.735 | |
| Race White (estimates) |  | |  | |  | |  | |  | |  | |  | |  | |  | | 59.71 | |
| (P value) |  | |  | |  | |  | |  | |  | |  | |  | |  | | 0.323 | |
| Constant | 4.138 | | 382.2 | | 0.73 | | 121.4 | | 3.571 | | 255.9 | | 4.321 | | 84.71 | | 0.932 | | 100.5 | |
| (P value) | 0 | | 0 | | 0.178 | | 0.005 | | 0 | | 0 | | 1E-05 | | 0 | | 0.276 | | 0.083 | |
| Observations | 58 | | 58 | | 54 | | 54 | | 79 | | 79 | | 45 | | 45 | | 46 | | 46 | |
| R^2^ | 0.293 | | 0.146 | | 0.044 | | 0.164 | | 0 | | 0 | | 0.148 | | 0.078 | | 0.057 | | 0.137 | |
| Adjusted R^2^ | 0.254 | | 0.115 | | 0.026 | | 0.131 | | 0 | | 0 | | 0.107 | | 0.056 | | 0.035 | | 0.076 | |
| Residual Std. Error | 0.378 | | 98.12 | | 0.851 | | 61.15 | | 0.458 | | 66.43 | | 1.078 | | 58.7 | | 1.051 | | 97.9 | |
| F Statistic | 7.469 | | 4.703 | | 2.404 | | 4.992 | |  | |  | | 3.638 | | 3.629 | | 2.646 | | 2.231 | |

| **Table S5.** Linear model estimates and P values per time of SARS-CoV-2 infection by sample type | | | | | | | | | | | | | | | | | | | |  |
| --- | --- | --- | --- | --- | --- | --- | --- | --- | --- | --- | --- | --- | --- | --- | --- | --- | --- | --- | --- | --- |
|  |  | | | |  | |  | |  | |  | |  | |  | |  | |  | |
| *Independent variables:* | *Dependent variables:* | | | | | | | | | | | | | | | | | | | |
|  | **Mother gut** | | | | **Mother vagina** | | | | **Mother tongue** | | | | **Infant gut** | | | | **Infant tongue** | | | |
|  | Shannon | | Chao1 | | Shannon | | Chao1 | | Shannon | | Chao1 | | Shannon | | Chao1 | | Shannon | | Chao1 | |
| SARS-CoV-2 at delivery-HC | 0.329 | | 77.37 | |  | | 65.162 | |  | |  | |  | |  | |  | |  | |
| (P value) | 0.03 | | 0.033 | |  | | 0.009 | |  | |  | |  | |  | |  | |  | |
| SARS-CoV-2 at delivery-Late | -0.039 | | -2.45 | |  | | 15.365 | |  | |  | |  | |  | |  | |  | |
| (P value) | 0.782 | | 0.944 | |  | | 0.5 | |  | |  | |  | |  | |  | |  | |
| SARS-CoV-2 at delivery-Active | 0.067 | | 73.91 | |  | | -0.512 | |  | |  | |  | |  | |  | |  | |
| (P value) | 0.638 | | 0.036 | |  | | 0.983 | |  | |  | |  | |  | |  | |  | |
| Gestational diabetes y | 0.462 | | 75.58 | |  | |  | |  | |  | |  | | 49.04 | |  | |  | |
| (P value) | 0.001 | | 0.02 | |  | |  | |  | |  | |  | | 0.064 | |  | |  | |
| Antibiotic during delivery y | 0.151 | |  | |  | |  | |  | |  | |  | |  | |  | |  | |
| (P value) | 0.169 | |  | |  | |  | |  | |  | |  | |  | |  | |  | |
| Pre pregnancy BMI |  | |  | | 0.026 | | 1.87 | |  | |  | | -0.052 | |  | |  | |  | |
| (P value) |  | |  | | 0.128 | | 0.134 | |  | |  | | 0.032 | |  | |  | |  | |
| Delivery mode Vaginal | |  | |  | |  | |  | |  | | -0.72 | |  | |  | |  | |  |
| (P value) |  | |  | |  | |  | |  | |  | | 0.059 | |  | |  | |  | |
| Infant weight g |  | |  | |  | |  | |  | |  | | 0.0005 | |  | |  | |  | |
| (P value) |  | |  | |  | |  | |  | |  | | 0.069 | |  | |  | |  | |
| Mother age |  | |  | |  | |  | |  | |  | |  | |  | | 0.042 | |  | |
| (P value) |  | |  | |  | |  | |  | |  | |  | |  | | 0.111 | |  | |
| Race Black |  | |  | |  | |  | |  | |  | |  | |  | |  | | -9.48 | |
| (P value) |  | |  | |  | |  | |  | |  | |  | |  | |  | | 0.892 | |
| Race Other (Hispanics) |  | |  | |  | |  | |  | |  | |  | |  | |  | | -21.7 | |
| (P value) |  | |  | |  | |  | |  | |  | |  | |  | |  | | 0.735 | |
| Race White |  | |  | |  | |  | |  | |  | |  | |  | |  | | 59.71 | |
| (P value) |  | |  | |  | |  | |  | |  | |  | |  | |  | | 0.323 | |
| Constant | 3.823 | | 306.1 | | 0.73 | | 56.284 | | 3.571 | | 255.9 | | 4.321 | | 84.71 | | 0.932 | | 100.5 | |
| (P value) | 0 | | 0 | | 0.178 | | 0.182 | | 0 | | 0 | | 1E-05 | | 0 | | 0.276 | | 0.083 | |
| Observations | 58 | | 58 | | 54 | | 54 | | 79 | | 79 | | 45 | | 45 | | 46 | | 46 | |
| R^2^ | 0.3 | | 0.234 | | 0.044 | | 0.173 | | 0 | | 0 | | 0.148 | | 0.078 | | 0.057 | | 0.137 | |
| Adjusted R^2^ | 0.233 | | 0.176 | | 0.026 | | 0.106 | | 0 | | 0 | | 0.107 | | 0.056 | | 0.035 | | 0.076 | |
| Residual Std. Error | 0.383 | | 94.69 | | 0.851 | | 62.02 | | 0.458 | | 66.43 | | 1.07 | | 58.7 | | 1.051 | | 97.9 | |
| F Statistic | 4.462 | | 4.039 | | 2.404 | | 2.569 | |  | |  | | 3.638 | | 3.62 | | 2.646 | | 2.23 | |

| **Table S6.** PERMANOVA parameters for SARS-CoV-2 infection or per time of SARS-CoV-2 infection and covariables for all body sites for pregnant women. P value for babies correspond only to those born vaginally. | | | | | | | |
| --- | --- | --- | --- | --- | --- | --- | --- |
| **Analysis** | **Group** | **Body site** | **Metric** | **Variables** | **R^2^ PERMANOVA** | **P value PERMANOVA** | **P value BETADISPER (sample size bias correction)** |
| By SARS-CoV-2 infection | Mother | Gut (Anal swab) | bray | COVID_diagnosis | 0.016 | 0.552 | 0.298 |
|  | Mother | Gut (Anal swab) | bray | Mother_age | 0.017 | 0.500 | NA |
|  | Mother | Gut (Anal swab) | bray | Pre_preg_BMI | 0.026 | 0.054 | NA |
|  | Mother | Gut (Anal swab) | bray | race | 0.046 | 0.811 | NA |
|  | Mother | Gut (Anal swab) | bray | gestational_diabetes | 0.021 | 0.192 | NA |
|  | Mother | Gut (Anal swab) | bray | antibiotic_during_delivery_y_n | 0.018 | 0.352 | NA |
|  | Mother | Gut (Anal swab) | Sorensen | COVID_diagnosis | 0.020 | 0.137 | 0.171 |
|  | Mother | Gut (Anal swab) | Sorensen | Mother_age | 0.018 | 0.343 | NA |
|  | Mother | Gut (Anal swab) | Sorensen | Pre_preg_BMI | 0.028 | 0.013 | NA |
|  | Mother | Gut (Anal swab) | Sorensen | race | 0.049 | 0.685 | NA |
|  | Mother | Gut (Anal swab) | Sorensen | gestational_diabetes | 0.020 | 0.172 | NA |
|  | Mother | Gut (Anal swab) | Sorensen | antibiotic_during_delivery_y_n | 0.017 | 0.441 | NA |
|  | Mother | Vagina | bray | COVID_diagnosis | 0.015 | 0.471 | 0.593 |
|  | Mother | Vagina | bray | Mother_age | 0.021 | 0.288 | NA |
|  | Mother | Vagina | bray | Pre_preg_BMI | 0.011 | 0.702 | NA |
|  | Mother | Vagina | bray | race | 0.043 | 0.682 | NA |
|  | Mother | Vagina | bray | gestational_diabetes | 0.011 | 0.685 | NA |
|  | Mother | Vagina | bray | antibiotic_during_delivery_y_n | 0.054 | 0.026 | NA |
|  | Mother | Vagina | Sorensen | COVID_diagnosis | 0.026 | 0.019 | 0.027 |
|  | Mother | Vagina | Sorensen | Mother_age | 0.016 | 0.717 | NA |
|  | Mother | Vagina | Sorensen | Pre_preg_BMI | 0.024 | 0.074 | NA |
|  | Mother | Vagina | Sorensen | race | 0.056 | 0.402 | NA |
|  | Mother | Vagina | Sorensen | gestational_diabetes | 0.021 | 0.193 | NA |
|  | Mother | Vagina | Sorensen | antibiotic_during_delivery_y_n | 0.026 | 0.020 | NA |
|  | Mother | Tongue | bray | COVID_diagnosis | 0.011 | 0.586 | 0.419 |
|  | Mother | Tongue | bray | Mother_age | 0.021 | 0.043 | NA |
|  | Mother | Tongue | bray | Pre_preg_BMI | 0.015 | 0.200 | NA |
|  | Mother | Tongue | bray | race | 0.042 | 0.224 | NA |
|  | Mother | Tongue | bray | gestational_diabetes | 0.017 | 0.137 | NA |
|  | Mother | Tongue | bray | antibiotic_during_delivery_y_n | 0.015 | 0.220 | NA |
|  | Mother | Tongue | Sorensen | COVID_diagnosis | 0.019 | 0.022 | 0.313 |
|  | Mother | Tongue | Sorensen | Mother_age | 0.017 | 0.048 | NA |
|  | Mother | Tongue | Sorensen | Pre_preg_BMI | 0.011 | 0.754 | NA |
|  | Mother | Tongue | Sorensen | race | 0.047 | 0.025 | NA |
|  | Mother | Tongue | Sorensen | gestational_diabetes | 0.012 | 0.491 | NA |
|  | Mother | Tongue | Sorensen | antibiotic_during_delivery_y_n | 0.019 | 0.012 | NA |
|  | Infant | Gut (Meconium) | bray | COVID_diagnosis | 0.027 | 0.192 | 0.709 |
|  | Infant | Gut (Meconium) | bray | Mother_age | 0.023 | 0.309 | NA |
|  | Infant | Gut (Meconium) | bray | Pre_preg_BMI | 0.042 | 0.025 | NA |
|  | Infant | Gut (Meconium) | bray | race | 0.071 | 0.258 | NA |
|  | Infant | Gut (Meconium) | bray | gestational_diabetes | 0.019 | 0.526 | NA |
|  | Infant | Gut (Meconium) | bray | antibiotic_during_delivery_y_n | 0.023 | 0.294 | NA |
|  | Infant | Gut (Meconium) | bray | delivery_mode | 0.047 | 0.010 | NA |
|  | Infant | Gut (Meconium) | Sorensen | COVID_diagnosis | 0.027 | 0.105 | 0.075 |
|  | Infant | Gut (Meconium) | Sorensen | Mother_age | 0.022 | 0.491 | NA |
|  | Infant | Gut (Meconium) | Sorensen | Pre_preg_BMI | 0.030 | 0.027 | NA |
|  | Infant | Gut (Meconium) | Sorensen | race | 0.061 | 0.857 | NA |
|  | Infant | Gut (Meconium) | Sorensen | gestational_diabetes | 0.025 | 0.219 | NA |
|  | Infant | Gut (Meconium) | Sorensen | antibiotic_during_delivery_y_n | 0.025 | 0.171 | NA |
|  | Infant | Gut (Meconium) | Sorensen | delivery_mode | 0.021 | 0.641 | NA |
|  | Infant | Tongue | bray | COVID_diagnosis | 0.039 | 0.021 | 0.141 |
|  | Infant | Tongue | bray | Mother_age | 0.012 | 0.982 | NA |
|  | Infant | Tongue | bray | Pre_preg_BMI | 0.021 | 0.547 | NA |
|  | Infant | Tongue | bray | race | 0.069 | 0.383 | NA |
|  | Infant | Tongue | bray | gestational_diabetes | 0.022 | 0.475 | NA |
|  | Infant | Tongue | bray | antibiotic_during_delivery_y_n | 0.013 | 0.962 | NA |
|  | Infant | Tongue | bray | delivery_mode | 0.019 | 0.703 | NA |
|  | Infant | Tongue | Sorensen | COVID_diagnosis | 0.026 | 0.124 | 0.400 |
|  | Infant | Tongue | Sorensen | Mother_age | 0.019 | 0.804 | NA |
|  | Infant | Tongue | Sorensen | Pre_preg_BMI | 0.025 | 0.194 | NA |
|  | Infant | Tongue | Sorensen | race | 0.071 | 0.251 | NA |
|  | Infant | Tongue | Sorensen | gestational_diabetes | 0.021 | 0.595 | NA |
|  | Infant | Tongue | Sorensen | antibiotic_during_delivery_y_n | 0.022 | 0.544 | NA |
|  | Infant | Tongue | Sorensen | delivery_mode | 0.018 | 0.921 | NA |
| By time of SARS-CoV-2 infection | Mother | Gut (Anal swab) | bray | Time of SARS-CoV-2 infection | 0.052 | 0.449 | 0.348 |
|  | Mother | Gut (Anal swab) | bray | Mother_age | 0.017 | 0.441 | NA |
|  | Mother | Gut (Anal swab) | bray | Pre_preg_BMI | 0.026 | 0.045 | NA |
|  | Mother | Gut (Anal swab) | bray | race | 0.048 | 0.686 | NA |
|  | Mother | Gut (Anal swab) | bray | gestational_diabetes | 0.020 | 0.196 | NA |
|  | Mother | Gut (Anal swab) | bray | antibiotic_during_delivery_y_n | 0.018 | 0.385 | NA |
|  | Mother | Gut (Anal swab) | Sorensen | Time of SARS-CoV-2 infection | 0.060 | 0.079 | 0.557 |
|  | Mother | Gut (Anal swab) | Sorensen | Mother_age | 0.018 | 0.320 | NA |
|  | Mother | Gut (Anal swab) | Sorensen | Pre_preg_BMI | 0.028 | 0.014 | NA |
|  | Mother | Gut (Anal swab) | Sorensen | race | 0.052 | 0.388 | NA |
|  | Mother | Gut (Anal swab) | Sorensen | gestational_diabetes | 0.019 | 0.206 | NA |
|  | Mother | Gut (Anal swab) | Sorensen | antibiotic_during_delivery_y_n | 0.018 | 0.311 | NA |
|  | Mother | Vagina | bray | Time of SARS-CoV-2 infection | 0.037 | 0.835 | 0.629 |
|  | Mother | Vagina | bray | Mother_age | 0.021 | 0.295 | NA |
|  | Mother | Vagina | bray | Pre_preg_BMI | 0.011 | 0.741 | NA |
|  | Mother | Vagina | bray | race | 0.045 | 0.672 | NA |
|  | Mother | Vagina | bray | gestational_diabetes | 0.011 | 0.721 | NA |
|  | Mother | Vagina | bray | antibiotic_during_delivery_y_n | 0.060 | 0.030 | NA |
|  | Mother | Vagina | Sorensen | Time of SARS-CoV-2 infection | 0.086 | 0.001 | 0.218 |
|  | Mother | Vagina | Sorensen | Mother_age | 0.016 | 0.696 | NA |
|  | Mother | Vagina | Sorensen | Pre_preg_BMI | 0.024 | 0.046 | NA |
|  | Mother | Vagina | Sorensen | race | 0.054 | 0.516 | NA |
|  | Mother | Vagina | Sorensen | gestational_diabetes | 0.020 | 0.217 | NA |
|  | Mother | Vagina | Sorensen | antibiotic_during_delivery_y_n | 0.023 | 0.069 | NA |
|  | Mother | Tongue | bray | Time of SARS-CoV-2 infection | 0.032 | 0.800 | 0.530 |
|  | Mother | Tongue | bray | Mother_age | 0.021 | 0.036 | NA |
|  | Mother | Tongue | bray | Pre_preg_BMI | 0.015 | 0.201 | NA |
|  | Mother | Tongue | bray | race | 0.041 | 0.283 | NA |
|  | Mother | Tongue | bray | gestational_diabetes | 0.017 | 0.128 | NA |
|  | Mother | Tongue | bray | antibiotic_during_delivery_y_n | 0.016 | 0.178 | NA |
|  | Mother | Tongue | Sorensen | Time of SARS-CoV-2 infection | 0.045 | 0.046 | 0.114 |
|  | Mother | Tongue | Sorensen | Mother_age | 0.017 | 0.069 | NA |
|  | Mother | Tongue | Sorensen | Pre_preg_BMI | 0.011 | 0.761 | NA |
|  | Mother | Tongue | Sorensen | race | 0.047 | 0.022 | NA |
|  | Mother | Tongue | Sorensen | gestational_diabetes | 0.012 | 0.499 | NA |
|  | Mother | Tongue | Sorensen | antibiotic_during_delivery_y_n | 0.018 | 0.026 | NA |
|  | Infant | Gut (Meconium) | bray | Time of SARS-CoV-2 infection | 0.063 | 0.520 | 0.751 |
|  | Infant | Gut (Meconium) | bray | Mother_age | 0.025 | 0.272 | NA |
|  | Infant | Gut (Meconium) | bray | Pre_preg_BMI | 0.040 | 0.030 | NA |
|  | Infant | Gut (Meconium) | bray | race | 0.071 | 0.278 | NA |
|  | Infant | Gut (Meconium) | bray | gestational_diabetes | 0.018 | 0.621 | NA |
|  | Infant | Gut (Meconium) | bray | antibiotic_during_delivery_y_n | 0.022 | 0.406 | NA |
|  | Infant | Gut (Meconium) | bray | delivery_mode | 0.045 | 0.012 | NA |
|  | Infant | Gut (Meconium) | Sorensen | Time of SARS-CoV-2 infection | 0.069 | 0.377 | 0.729 |
|  | Infant | Gut (Meconium) | Sorensen | Mother_age | 0.021 | 0.664 | NA |
|  | Infant | Gut (Meconium) | Sorensen | Pre_preg_BMI | 0.030 | 0.020 | NA |
|  | Infant | Gut (Meconium) | Sorensen | race | 0.062 | 0.850 | NA |
|  | Infant | Gut (Meconium) | Sorensen | gestational_diabetes | 0.024 | 0.269 | NA |
|  | Infant | Gut (Meconium) | Sorensen | antibiotic_during_delivery_y_n | 0.026 | 0.138 | NA |
|  | Infant | Gut (Meconium) | Sorensen | delivery_mode | 0.021 | 0.654 | NA |
|  | Infant | Tongue | bray | Time of SARS-CoV-2 infection | 0.116 | 0.002 | 0.924 |
|  | Infant | Tongue | bray | Mother_age | 0.015 | 0.871 | NA |
|  | Infant | Tongue | bray | Pre_preg_BMI | 0.020 | 0.540 | NA |
|  | Infant | Tongue | bray | race | 0.065 | 0.449 | NA |
|  | Infant | Tongue | bray | gestational_diabetes | 0.020 | 0.521 | NA |
|  | Infant | Tongue | bray | antibiotic_during_delivery_y_n | 0.013 | 0.961 | NA |
|  | Infant | Tongue | bray | delivery_mode | 0.019 | 0.666 | NA |
|  | Infant | Tongue | Sorensen | Time of SARS-CoV-2 infection | 0.079 | 0.015 | 0.698 |
|  | Infant | Tongue | Sorensen | Mother_age | 0.021 | 0.650 | NA |
|  | Infant | Tongue | Sorensen | Pre_preg_BMI | 0.023 | 0.365 | NA |
|  | Infant | Tongue | Sorensen | race | 0.070 | 0.238 | NA |
|  | Infant | Tongue | Sorensen | gestational_diabetes | 0.019 | 0.804 | NA |
|  | Infant | Tongue | Sorensen | antibiotic_during_delivery_y_n | 0.022 | 0.465 | NA |
|  | Infant | Tongue | Sorensen | delivery_mode | 0.018 | 0.889 | NA |

| **Table S7.** Vaginal Community State Type (CST) prevalence among SARS-CoV-2 infections during pregnancy. CST describes the dominance or lack thereof of *Lactobacillus* species of the vaginal microbiota. | | | | | | |
| --- | --- | --- | --- | --- | --- | --- |
|  |  |  |  |  |  |  |
|  | **SARS-CoV-2 positive pregnant women (N=43)** | | | **Healthy controls pregnant women (HC, N=11)** | **TOTAL (N=54)** | ***P value &*** |
|  | **Early infection (N=18)** | **Late infection (N=13)** | **Active infection (N=12)** |  |  |  |
| **CST** |  |  |  |  |  | 0.913 |
| I (*L. crispatus*) | 7 (38.9%) | 5 (38.5%) | 6 (50.0%) | 5 (45.5%) | 23 (42.6%) | 0.945 |
| II (*L. gasseri*) | 0 (0%) | 0 (0%) | 0 (0%) | 1 (9.1%) | 1 (1.9%) | 0.783 |
| III (*L. iners*) | 7 (38.9%) | 5 (38.5%) | 6 (50.0%) | 4 (36.4%) | 22 (40.7%) | 0.927 |
| IV (Non-*Lactobacillus* dominance) | 3 (16.7%) | 2 (15.4%) | 0 (0%) | 1 (9.1%) | 6 (11.1%) | 0.866 |
| V (*L. jensenii*) | 1 (5.6%) | 1 (7.7%) | 0 (0%) | 0 (0%) | 2 (3.7%) | 0.634 |
| & Fisher's exact test | | | | |  |  |
